# Pharmacokinetics and Pharmacodynamics of Key Components of a Standardized *Centella asiatica* Product in Cognitively Impaired Older Adults: A Phase 1, Double-Blind, Randomized Clinical Trial

**DOI:** 10.3390/antiox11020215

**Published:** 2022-01-23

**Authors:** Kirsten M. Wright, Melissa Bollen, Jason David, Alex B. Speers, Mikah S. Brandes, Nora E. Gray, Armando Alcázar Magaña, Christine McClure, Jan F. Stevens, Claudia S. Maier, Joseph F. Quinn, Amala Soumyanath

**Affiliations:** 1 Department of Neurology, Oregon Health & Science University, Portland, OR 97239, USA; wrigkir@ohsu.edu (K.M.W.); bollen@ohsu.edu (M.B.); jasondavid182@gmail.com (J.D.); speers@ohsu.edu (A.B.S.); brandes@ohsu.edu (M.S.B.); grayn@ohsu.edu (N.E.G.); mcclurch@ohsu.edu (C.M.); quinnj@ohsu.edu (J.F.Q.); 2 Department of Chemistry, Oregon State University, Corvallis, OR 97331, USA; alcazara@oregonstate.edu (A.A.M.); claudia.maier@oregonstate.edu (C.S.M.); 3 Department of Pharmaceutical Sciences, Oregon State University, Corvallis, OR 97331, USA; fred.stevens@oregonstate.edu; 4 Linus Pauling Institute, Oregon State University, Corvallis, OR 97331, USA; 5 Department of Neurology, Veterans Affairs Portland Health Care System Center, Portland, OR 97239, USA

**Keywords:** *Centella asiatica*, pharmacokinetics, pharmacodynamics, *NRF2*, antioxidant, Alzheimer’s disease, tolerability

## Abstract

*Centella asiatica* is reputed in Eastern medicine to improve cognitive function in humans. Preclinical studies have demonstrated that aqueous extracts of *C. asiatica* improve cognition in mouse models of aging and Alzheimer’s disease (AD) through the modulation of mitochondrial biogenesis and nuclear factor-erythroid-2-related factor 2 (*Nrf2)*-dependent antioxidant response genes. This randomized, double-blind, crossover Phase I trial explored the oral bioavailability and pharmacokinetics of key compounds from two doses (2 g and 4 g) of a standardized *C. asiatica* aqueous extract product (CAP), over 10 h, in four mildly demented older adults on cholinesterase inhibitor therapy. The analysis focused on triterpenes (TTs) and caffeoylquinic acids (CQAs), which are known to contribute to *C. asiatica*’s neurological activity. The acute safety of CAP and the effects on *NRF2* gene expression in peripheral blood mononuclear cells were evaluated. Single administration of 2 g or 4 g of CAP was safe and well-tolerated. The TT aglycones, asiatic acid and madecassic acid, were identified in plasma and urine, while the parent glycosides, asiaticoside and madecassoside, although abundant in CAP, were absent in plasma and had limited renal excretion. Similarly, mono- and di-CQAs showed delayed absorption and limited presence in plasma or urine, while the putative metabolites of these compounds showed detectable plasma pharmacokinetic profiles and urinary excretion. CAP elicited a temporal change in *NRF2* gene expression, mirroring the TT aglycone’s pharmacokinetic curve in a paradoxical dose-dependent manner. The oral bioavailability of active compounds or their metabolites, *NRF2* target engagement, and the acute safety and tolerability of CAP support the validity of using CAP in future clinical studies.

## 1. Introduction

Alzheimer’s disease (AD) is a severe form of cognitive impairment and one of the most expensive and debilitating conditions known to modern medicine. In the United States (US), greater than six million people have AD, making it the sixth leading cause of death [1] with a projected annual healthcare cost in 2021 of USD 355 billion [2]. The prevalence of AD worldwide is estimated to be as high as 55 million and is expected to rise to 139 million by 2050, due to a global increase in the proportion of older adults in the population [3]. The pathogenesis of AD is highly complex. Current evidence suggests it is a combination of genetics, environment, and lifestyle factors, making the development of treatments very challenging. The most striking pathognomonic feature of AD within the brain is the accumulation of β-amyloid (Aβ) plaques and neurofibrillary tangles caused by the abnormal adhesion of tau proteins in neurons [4]. It is hypothesized that these plaques and tangles accumulate within and between neurons, subsequently disrupting normal cell function and synaptic communication and causing apoptosis [5].

Other key pathologic changes noted in AD are mitochondrial dysfunction and oxidative stress. Decreased expression and activity of key mitochondrial enzymes lead to reduced electron transport chain complex activity and ATP-synthesis, as well as impaired oxidative phosphorylation, contributing to an increase in free radicals and the prevalence of oxidative damage seen in AD [6,7]. Shifts in glucose metabolism, potentially due to decreased expression of glycolytic enzymes, from glucose to alternative ketone bodies as a fuel source and a lack of calcium ion homeostasis also contribute to increased oxidative stress and eventual neuronal death in AD [6,7,8,9]. In this context, the nuclear factor-erythroid- 2-related factor 2 (NRF2) has been identified as a potential therapeutic target in AD, as this factor regulates the expression of antioxidant response genes and modulates mitochondrial function and biogenesis [10].

The US Food and Drug Administration (FDA) approved drugs currently used for the symptomatic treatment of AD are cholinesterase inhibitors [11], *N*-methyl-d-aspartate (NMDA) receptor antagonists [12,13], and one anti-amyloid immunotherapy (aducanumab) [14]. Unfortunately, their effectiveness is apparent only in the mild stages of cognitive impairment and is highly variable. Current pharmaceutical investigation is aimed at preventing the accumulation of, or promoting the clearance of, neurotoxic Aβ plaques (anti-amyloid immunotherapy) [15,16]; however, recent trials of such agents have only shown efficacy in mild cognitive impairment (MCI), have failed to produce a significant clinical effect on AD, and have demonstrated some significant adverse effects [17,18]. Beyond cognitive impairment, AD also has significant comorbidities including agitation, insomnia, depression [19] and anxiety [20]. Multiple interventions are often needed to manage these symptoms, thereby affecting patient compliance and safety. There is an acknowledged need [21] to develop or identify novel disease-modifying agents to prevent progressive cognitive decline and treat AD’s comorbidities; patients and healthcare providers currently have limited pharmaceutical options for this debilitating disease.

*Centella asiatica*, also known as gotu kola [22], is a viable candidate for development as a rational phytotherapeutic agent for cognitive decline and AD. *C. asiatica* has a long history in Ayurvedic medicine as a rejuvenative herb that improves memory and brain function [23,24]. Preclinical studies have shown that aqueous extracts of *C. asiatica* (CAW) have important biological effects on aging, mood, learning, memory, and, potentially, AD development [25]. In preclinical models, CAW was found to mediate the impact of oxidative stress, which is implicated in cognitive decline and AD, thereby preventing cognitive deficits [26,27,28]. The mechanisms through which CAW shows such remarkable cognitive enhancing and neuroprotective properties include the modulation of mitochondrial biogenesis and the activation of antioxidant response genes [29]. These properties of CAW appear to be linked through its ability to activate *Nrf2 expression* [30,31]. There is limited research on the effects of *C. asiatica* on cognition and neurological disorders in humans, but the existing human studies show improved cognition, mood, and quality of life [23]. These studies also highlight the significant heterogeneity in the types of interventions used, the doses selected, the levels of standardization, and the populations studied, making translation into wider clinical use challenging [23].

**Figure 1:**
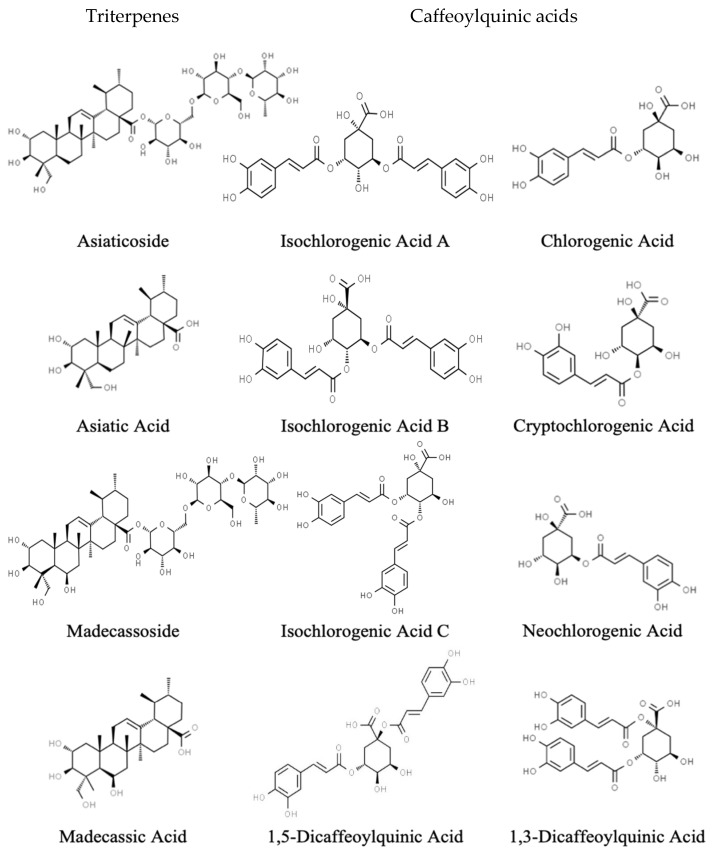
Structures of the triterpenoid saponins, aglycones, monocaffeoylquinic acids, and dicaffeoylquinic acids identified in *Centella asiatica* water extract.

Chemical analyses of *C. asiatica* have identified unique specialized metabolites, triterpenoid (TT) saponin glycosides (asiaticoside and madecassoside), and their aglycones (asiatic acid and madecassic acid) [32,33], which have been associated with *C. asiatica’s* neuroprotective and neurotropic effects (Figure 1) [34,35,36,37]. Mouse studies have shown improvements in cognition in wild-type and AD models using crude water extracts with or without TT aglycones [29,38], which suggests that there are other neuroactive ingredients within CAW. Our group has found, using high-performance liquid chromatography coupled to mass spectral detection (HPLC-MS), additional active phenolic components, known as caffeoylquinic acids (CQAs), in CAW (Figure 1) [39]. Studies have shown that mono-CQAs protect primary cortical neurons from glutamate excitotoxicity in mice [23,40], while di-CQAs protect rat cortical neurons from cell death, excitotoxic and hypoxic damage [41], reactive oxygen species, and glutamate-induced increases in calcium [23,41]. We have previously identified that CQAs protect against Aβ-induced cytotoxicity [39], increase *Nrf2* gene expression [30], increase mitochondrial respiration in neuroblastoma cells [39], and improve cognition in the 5xFAD mouse model of AD [27]. Such evidence strongly supports the hypothesis that CQAs have strong neuroprotective properties, contribute to the biological activity of *C. asiatica*, and would be valuable compounds as a treatment option for AD and cognitive decline.

Interest in the potential pharmacological properties of *C. asiatica* has led to a variety of pharmacodynamic studies, but there is a notable lack of human pharmacokinetic data [42,43]. Of the available studies, most have focused on the TTs (most specifically asiatic acid) using purified extracts or isolated compounds [42,44,45] and not on complete extracts prepared using methods more similar to the traditional methods. The use of purified test materials eliminates possible chemical interactions and bioactive synergistic compounds that may occur in crude extract preparations. In the present study, we explored the pharmacokinetics of both the TT and CQA components of the *C. asiatica* crude water extract, CAW, in elderly humans with MCI. CAW was administered at two doses (2 g and 4 g) in a formulated product (CAP) [46] that was standardized for the content of the TT and CQA components shown in Figure 1. *NRF2* activation in humans following CAP administration was also investigated. This is the first study to consider the pharmacokinetics of both the TTs and CQAs from *C. asiatica* and to use a complex extract as the source of these compounds.

## 2. Materials and Methods

### 2.1. Chemicals

For plasma and urine sample work-up, preparation of calibration standards, and high-performance liquid-chromatography-tandem mass spectrometry (HPLC-MS/MS) analysis, methanol (HPLC grade), *Aerobacter aerogenes* sulfatase (10–20 units/mL), *Escherichia coli* glucuronidase (5000–50,000 units/mL), asiatic acid, caffeic acid, cryptochlorogenic acid, 1,5-dicaffeoylquinic acid, ferulic acid, and ^13^C_3_-ferulic acid were purchased from Sigma Aldrich (Darmstadt, Germany). Acetonitrile (HPLC-MS grade), L-ascorbic acid, and glycerol were purchased from Fisher Scientific (Fairlawn, NJ, USA). Formic acid (HPLC-MS grade) and internal standard chrysin were purchased from Honeywell Fluka (Charlotte, NC, USA) and de-ionized water (HPLC grade) was purchased from Macron Fine Chemicals (Radnor, PA, USA). TT compounds (asiaticoside, madecassic acid, and madecassoside) and caffeoylquinic acid compounds (1,4-dicaffeoylquinic acid, neochlorogenic acid, isochlorogenic acid A, and isochlorogenic acid B) were purchased from TransMIT (Gießen, Germany). Dihydroferulic acid and 3-(3-hydroxyphenyl)propionic acid (HPP) were purchased from Toronto Research Chemicals (Toronto, ON, Canada). d_3_-Isoferulic acid and d_3_-dihydroisoferulic acid were purchased from Santa Cruz Biotechnology (Dallas, TX, USA). Chlorogenic acid, 1,3-dicaffeoylquinic acid, dihydrocaffeic acid, isochlorogenic acid C, isoferulic acid, and internal standard ursolic acid were purchased from Chromadex (Irvine, CA, USA). Compound identity was verified using HPLC-MS/MS and a comparison against their expected retention time and m/z values. The purity of all compounds was 94.8–99.4%.

### 2.2. Centella Asiatica Water Extract Product (CAP)

**Figure 2:**
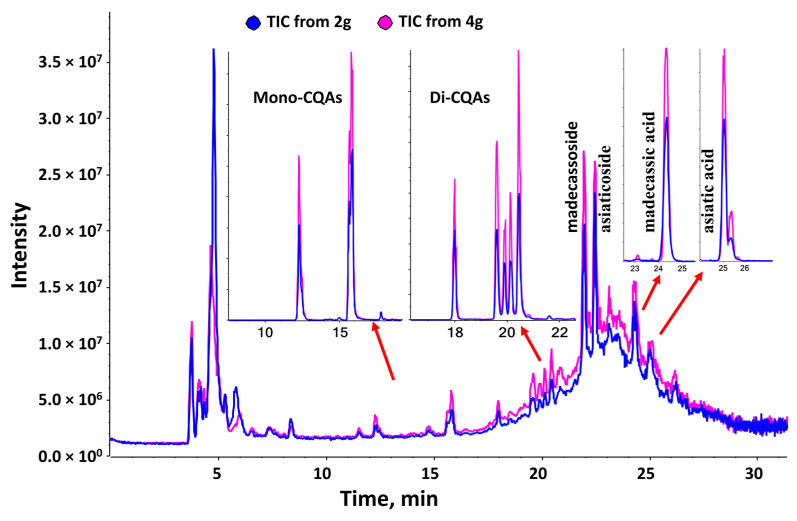
Total ion chromatogram (TIC) of *Centella asiatica* water extract product (CAP) showing peaks corresponding to the triterpene and caffeoylquinic acid components of interest. The total ion chromatogram in negative ion mode corresponds to 2 g (blue) and 4 g (pink) of the *Centella asiatica* water extract present in each sachet (~20 g). The major compounds are indicated in the chromatogram. Extracted ion chromatograms (XIC) are shown for the monocaffeoylquinic acids (mono-CQAs; *m*/*z* 353.08, [M-H]^−^), the dicaffeoylquinic acids (Di-CQA, *m*/*z* 515.12, [M-H]^−^), madecassic acid (*m*/*z* 503.3, [M-H]^−^), and asiatic acid (*m*/*z* 487.3, [M-H]^−^).

Allometric scaling [47] identified a dose range of 2–10g of CAW daily for a 70 kg human to be equivalent to the 200–1000 mg/kg/day estimated intake in mice in our preclinical studies where good biological activity was observed [10,27,29,34,38,39,48,49,50,51,52,53]. Two doses (2 g and 4 g) within the range were selected based upon the 200 mg/kg/d and 500 mg/kg/d doses that provided robust cognitive improvements in mice [27,29,38,49,50]. A large-scale dried aqueous extract of *C.* *asiatica* (CAW) was prepared using a method scaled up from our preclinical studies at Ashland laboratories (Kearny, NJ, USA), a certified Good Manufacturing Practice (cGMP) facility [46]. The CAW extract was spray-dried onto a carrier matrix at Ashland laboratories and blended at Oregon’s Wild Harvest (Redmond, OR, USA) with inert agents (excipients) imparting color and flavor, into a powdered formula called *C. asiatica* water extract product (CAP) [46]. The excipients served to improve the palatability and dispersibility in water for human consumption while imparting a color that could be matched in a placebo for future trials. The CAP products were standardized to contain identical levels of excipients in the 2 g and 4 g doses. Each dose was packaged into individual opaque sachets by Oregon’s Wild Harvest, labeled to prevent the unblinding of study personnel and participants, and shipped to the Oregon Health & Science University (OHSU) Research Pharmacy for storage and dispensing. In parallel, sachets of each dose (*n* = 5) were analyzed using HPLC-high-resolution tandem mass spectral (LC-HRMS/MS) fingerprinting using our previously published methodology [52,54] at Oregon State University (Corvallis, OR, USA) to confirm the presence of known bioactive compounds and the expected two-fold increase between the 2 g and 4 g doses (Table 1, Figure 2). Briefly, the content of each sachet (~20 g) was suspended in 100 mL of aqueous methanol (70% *v*/*v*) containing formic acid (0.1% *v*/*v*). The samples were sonicated at room temperature for 30 min, and an aliquot (1 mL) of the suspension was centrifuged (15,000 rpm for 10 min) and diluted 100 times before injection (10 µL) for HPLC-HRMS/MS. The product was stored in the dark at −20 °C for the duration of the study.

### 2.3. Ethical Statement and Informed Consent

This study protocol was approved by the OHSU Institutional Review Board (IRB number: 17985, date of approval: 24 May 2019) and was registered with the United States National Library of Medicine Clinical Trials Registry ClinicalTrials.gov (NCT03937908, date of first registration: 6 May 2019). The clinical study was conducted at OHSU in the Oregon Clinical and Translational Research Institute’s (OCTRI) Clinical Trials Research Center (Portland, OR, USA) in accordance with the Declaration of Helsinki. All participants provided written informed consent for inclusion prior to participation in the study. The study was performed under a US FDA Investigational New Drug assignment (#13066) for CAP.

### 2.4. Eligibility Criteria for Participants

Participants were recruited from October 2019 until March 2020, when all study activities were placed on hold due to the SARS-CoV-2-related restrictions on human subject research of vulnerable populations. Potential participants (*n* = 185) were identified using the Oregon Clinical and Translational Research Institute’s research volunteer repository, the National Institute of Aging’s Oregon Alzheimer’s Disease Research Center, the National Institute of Neurological Disorders and Stroke NeuroNEXT database, and through referrals from OHSU neurologists and gerontologists. Of these, a subset (*n* = 31) was evaluated by an examination of medical records and/or telephone screening. Five volunteers, aged 65–85 years, that met the National Institute on Aging and Alzheimer’s Association core clinical criteria for mild cognitive impairment (MCI) with a Clinical Dementia Rating score of 0.5–1 and a Mini Mental State Examination score of 20–28 were enrolled. The participants reported a history of subjective memory decline with a gradual onset and a slow progression for at least one year prior to enrollment that was not vascular dementia, normal pressure hydrocephalus, or Parkinson’s disease and was corroborated by an informant. All participants were on a stable dose of cholinesterase inhibitor therapy for at least 12 weeks prior to enrollment and had to remain on this dose for the duration of the study. Volunteers were excluded if they had any significant symptoms of depression, schizophrenia, or any other major psychiatric disorder; had a body mass index below 17 or above 35; were found to have an asymptomatic urinary tract infection; or had a history of cancer within five years of study onset, smoked tobacco, or had a diagnosis of alcohol or substance abuse. They were also excluded if they had any of the following comorbidities: diabetes mellitus, kidney failure, liver failure, hepatitis, blood disorders, clinical symptomatic orthostatic hypotension, unstable or significantly symptomatic cardiovascular disease, or a significant disease of the central nervous system, such as brain tumor, seizure disorder, subdural hematoma, cranial arteritis, or a clinically significant stroke. As this was a safety and bioavailability study, all participants on the following medication classes were excluded: sedatives, central nervous system active medications that have not been stable for two months, anticoagulants, investigational drugs used within five half-lives of the baseline visit, systemic corticosteroids, neuroleptics, anti-Parkinsonian agents, narcotic analgesics, nicotine, or *Cannabis sativa.*

### 2.5. Study Sample Size

To be able to compare the pharmacokinetic parameters between the CAP 2g and CAP 4g doses, the sample size was calculated based on changes in the response values from the literature [42] for two outcomes, peak concentration (C_max_) and bioavailability/area-under-the-curve (AUC), at two treatment doses. With respect to C_max_, estimations from G*Power indicated a sample size of six would be sufficient to detect the established criterion difference (critical t = 2.57). The post-treatment mean difference in C_max_ from the literature was found to be 0.66 and was replicated during simulation, with confidence intervals indicating a significant difference (0.36, 0.96). Both the observed mean and median test statistics were found to be greater than the critical t (mean t = 4.23, median t = 3.88). Mean power was found to be slightly lower than expected (84% versus 86%), although median power was significantly larger (92%). Accordingly, six subjects were deemed sufficient to detect a difference in C_max_ between the two doses of CAP. The target recruitment for this study was for 8 participants, 4 male and 4 female, allowing for 25% dropouts; however, pandemic-related regulations stopped recruitment before six subjects could be met and the study was terminated due to the associated fiscal limitations.

### 2.6. Study Design and Sample Collection

This was a single-center, randomized, double blind, crossover study of two acute dosages (2 g and 4 g) of CAP. The participants were randomized using an arm equivalence design, by the OHSU Research Pharmacy, to receive each dose of CAP in a different order to prevent tolerance effects and ensure some data was available from each dose in the case of a dropout after the first dose. All study personnel were blinded to the participant’s randomization until all data analysis had been completed. All participants consumed both doses of CAP and attended two study visits a minimum of 14 days apart. They were placed on a low phytochemical diet for 48 h prior to and during each study visit in order to minimize interference from dietary TTs and CQAs and ensure that any such compounds identified in the plasma and urine were derived from *C. asiatica*. To ensure compliance, all participants completed a diet diary over the course of the 48 h. The participants fasted for a minimum of 10 h prior to each study visit, excluding water, to standardize gastrointestinal transit time and minimize delayed absorption due to the presence of food.

In the morning of each study visit, a baseline blood sample (20 mL) was collected via a peripheral intravenous catheter. To maintain participant blinding, a member of the study personnel dissolved the allocated dose of CAP in 10 ounces of warm water and the participant consumed the study intervention orally as a single bolus. Blood samples (10 mL) were collected via the peripheral catheter over a 10 h post-administration period at 0.25, 0.5, 0.75, 1, 1.5, 2, 2.5, 3, 4, 6, 8, and 10 h. Blood was transferred to a BD Vacutainer™ heparinized tube (Fisher Scientific) and centrifuged for 10 min at 10,000× *g* and 4 °C to isolate plasma. Plasma was frozen in 2mL aliquots and kept at −80 °C until analysis. At the time points 0, 1, 2, 3, 4, and 6 h, an additional 5 mL of blood was collected for the isolation of peripheral blood mononuclear cells (PBMCs) and *NRF2* gene expression analysis. All participants were asked to refrain from consuming food until 2 h after consumption of the study intervention at each visit to prevent delayed absorption due to food effects. Breakfast (at 2 h following CAP ingestion), lunch, dinner and snacks, all meeting the low phytochemical criteria, were provided by the OCTRI Bionutrition Unit. Urine samples were collected at baseline and over the 10 h post-administration period at each visit to measure renal excretion. All post-administration urinary output was combined in a 2 L urine collection container stored at 4 °C during the study visit. The total amount of post CAP administration urine was recorded, and a 50 mL aliquot was frozen at −80 °C for analysis. At the second visit, participants consumed the dose they were not randomized to receive at the first visit and underwent all study procedures previously described.

### 2.7. Safety Assessments

Safety and acute tolerability assessments were performed during each study visit. A comprehensive metabolic panel was collected at baseline and at 10 h post-administration of CAP at each study visit to identify asymptomatic metabolic changes. An electrocardiogram was collected at baseline and six hours post administration to identify asymptomatic cardiovascular changes. At baseline, 10 h, 24 h, and 7 days post administration, a multi-system questionnaire was used to identify acute and delayed adverse events. Each event was ranked on a scale of 0–5 based upon the following criteria: 0 = absent, 1 = mild, 2 = moderate, 3 = severe, 4 = life-threatening, and 5 = fatal.

### 2.8. Sample Preparation and Pharmacokinetic Analysis

Sample preparation was performed using a protein precipitation method adapted from Cheng et al. [55] Duplicate plasma samples (50 µL) were combined with an ascorbic acid solution (1%; 10 µL) and incubated for 20 min at 37 °C using *A. aerogenes* sulfatase (2.5 µL; 0.025–0.05 units) and *E. coli glucuronidase* (2.5 µL; 12.5–125 units) dissolved in 50% aqueous glycerol to release the analytes from their sulfate and β-d-glucuronic acid esters. Proteins were precipitated by adding a crash solution (200 µL) consisting of 3:1 acetonitrile:methanol containing internal standards (20 µg/mL d_3_-isoferulic acid, d_3_-dihydroisoferulic acid, and chrysin or 50 µg/mL ^13^C_3_-ferulic acid and ursolic acid), holding for 30 min at 4 °C and then centrifuged for 5 min at 10,000× *g*. The supernatant was filtered through a 0.22 µm Ultrafree MC-GV spin filter (Millipore Sigma) at 10,000× *g* for 5 min at 4 °C and then transferred to vials for analysis. For TT analysis, the concentration of organic solvent (the sample filtrate) to water in each sample was adjusted to a 60:40 ratio to optimize the peak shape, and for CQA analysis, the concentration of organic solvent to 1% aqueous formic acid in each sample was adjusted to a 90:10 ratio to optimize the peak shape.

Due to the possibility of residual diet-derived interference and unique matrix effects in each participant’s plasma, a calibration curve was generated for each visit by spiking standards into the participant’s baseline plasma collected at that visit. All calibration samples (50 µL) were subjected to the method described above, except that a sham enzyme hydrolysis was performed using 50% aqueous glycerol (5 µL) instead of the enzyme solutions. Calibration standards were processed in parallel with the study samples being analyzed in the same HPLC-MS/MS run. The calibration curves showed good linearity for TTs (R^2^ = 0.93–0.99; 0–60 ng/mL for the glycosides and 0–450 ng/mL for the aglycones), CQAs (R^2^ = 0.99; 0–30 ng/mL), and putative CQA metabolites (R^2^ = 0.96–1; 0–30 ng/mL for all metabolites except 0–130 ng/mL for 3-(3-hydroxyphenyl)propionic acid).

For renal excretion analyses, urine was thawed and prepared using the same work-up methods as the plasma samples except for enzymatic digestion. To address the potential effect of phase II metabolism, all urine samples were incubated in triplicate with 50% glycerol (sham enzyme) or with enzymes as described for plasma, to measure unconjugated analyte and total analyte, respectively. Calibration curves were prepared in baseline urine and treated with sham enzyme.

Plasma and urine samples collected at the participant’s screening visit were analyzed in parallel with the samples collected from each study visit to determine the efficacy of the low phytochemical diet in reducing analyte levels from dietary sources. The analysis of the prepared plasma and urine samples was performed at the OHSU Bioanalytical Shared Resource/Pharmacokinetics Core Laboratory (Portland, OR). For detection of TT glycosides and aglycones, an analytical method adapted from Nair et al. [56] was used. HPLC-MS/MS using selected reaction monitoring was performed on an Applied Biosystems Q-Trap 4000 LC-MS instrument (Framingham, MA, USA). Chromatographic separation was achieved using a Poroshell 120 EC18 (3mm i.d. × 50 mm; 2.7 µ) column with a Poroshell 120 EC-C18 ultra high-performance liquid chromatography (UHPLC) (3.0 mm i.d. × 5 mm, 2.7 µ) guard column (Agilent; Santa Clara, CA, USA). The injection volume was 20 µL. Gradient elution was performed using a mobile phase of solvent A (water containing 10 mM ammonium acetate and 0.02% ammonium hydroxide; pH 8.5) and solvent B (methanol). The flow rate was 0.42 mL/min. The chromatographic method duration was 9 min, and the gradient design was as follows: an initial 2 min increase from 40–60% B, followed by 60–95% B from 2–3.5 min, hold at 95% B from 3.5–6 min, return to 40%B by 6.1 min, and re-equilibrate at 40% B from 6.1–9 min. TTs were detected as their ammonium adducts with positive ion mode electrospray ionization using the following MS/MS transitions (*m*/*z*): AA (506/453), MA (522/451), AS (976/453; 976/635), and MS (992/487; 992/451). The internal standard chrysin was detected as the molecular ion (255/255) and ursolic acid was detected as its ammonium adduct (474/411; 474/191). For the detection of CQAs and their associated metabolites, HPLC-MS/MS was performed on an Applied Biosystems 5500 QTRAP HPLC-MS instrument (Framingham, MA, USA). Chromatographic separation was achieved using a Zorbax Eclipse plus C8 Rapid resolution (4.6 i.d. × 150 mm, 3.5 µ) column with a Zorbax Eclipse plus C8 Rapid resolution (4.6 i.d. × 12.5 mm, 5 µ) guard column (Agilent, Santa Clara, CA, USA). The injection volume was 5 µL. Gradient elution was performed using a mobile phase of solvent A (water containing 0.05% *v*/*v* acetic acid) and solvent B (acetonitrile with 0.05% *v*/*v* acetic acid). The flow rate was 0.8 mL/min. The chromatographic method had a duration of 21 min, and the gradient design was as follows: an initial 0.1 min at 10% B, an increase to 25% B by 4.5 min, 25–40% B from 4.5–10 min, 40 to 95% B from 10–11 min, hold at 95% B from 11–16 min, return to 10% B by 16.2 min, and re-equilibrate at 10% from 16.2–21 min. All CQAs, metabolites, and internal standards were detected using negative ion mode electrospray ionization and the following MS/MS transitions (*m*/*z*): mono-CQAs (353/191); di-CQAs (515/353; 515/191); caffeic acid (179/135); ferulic acid and isoferulic acid (193/134); dihydrocaffeic acid (181/109); dihydroferulic acid and dihydroisoferulic acid (195/136), 3-(3-hydroxyphenyl)propionic acid (165/106), ^13^C_3_-ferulic acid (196/136), d_3_-isoferulic acid (196/134), and d_3_-dihydroisoferulic acid (198/136).

### 2.9. Sample Preparation and NRF2 Gene Expression Analysis

PBMCs were isolated using a BD Vacutainer™ CPT™ Mononuclear Cell Preparation Tube (Fisher Scientific). Briefly, samples were centrifuged for 20 min at 4 °C and 1800× *g* and the PBMC layer was isolated. PBMCs were rinsed in phosphate buffered saline (PBS) and centrifuged again at 250× *g* for 10 min. The resulting pellet was resuspended in Tri Reagent (Molecular Research Center, Cincinnati, OH, USA) and RNA was isolated per the manufacturer’s protocol. RNA was reverse transcribed with the Superscript III First Strand Synthesis kit (ThermoFisher, Waltham, MA, USA) to generate cDNA as per the manufacturer’s instructions. Relative mRNA expression was determined using TaqMan Gene Expression Master Mix (ThermoFisher, Waltham, MA, USA) and commercially available TaqMan primers (ThermoFisher, Waltham, MA, USA) for *NRF2 (NFE2L2)* and *GAPDH*. Quantitative PCR was performed on a QuantStudio3 Machine (Applied Biosystems, Waltham, MA, USA) and analyzed using the delta-delta Ct method normalizing to *GAPDH* expression.

### 2.10. Data Analysis

Analyst software from Sciex technologies was used to obtain the peak area for each analyte and internal standard. Totaling the peak area for three mono-CQAs and six di-CQAs was found to give improved area and area ratio calibration curves compared to individual curves for each compound, possibly due to the interconversion of isomers [57,58]. The areas for each compound group were also totaled prior to calculating the pharmacokinetic parameters. The pharmacokinetic parameters (AUC_0-10_, t_1/2_, T_max_, and C_max_) and the time curves were calculated using a non-compartmental analysis of plasma concentration versus time data using Excel software PK-solver (version 2.0). Two-sided paired t-tests were used to compare the pharmacokinetic and clearance parameters between the 2 g and 4 g doses. A *p*-value of less than 0.05 was considered statistically significant.

## 3. Results

### 3.1. Participant Baseline Clinical Characteristics

**Figure 3:**
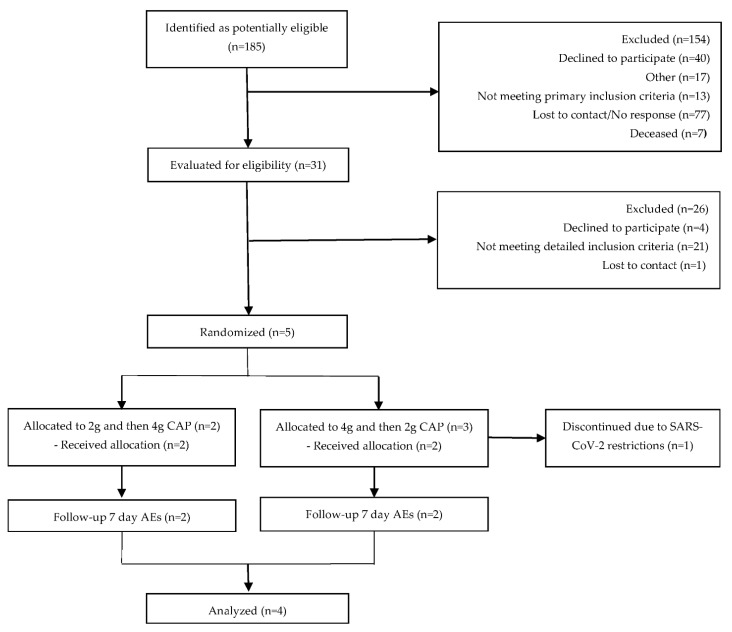
Consort flow diagram.

Participant characteristics and demographics are summarized in Table 2. The age of the five enrolled participants was between 67 and 77 years. All participants were on donepezil, taking 10 mg twice daily (*n* = 1), 10 mg daily (*n* = 3), or 5 mg daily (*n* = 1) for an average of 3.1 ± 2 years. One participant was discontinued prior to the first study visit (Figure 3) due to institutional regulations stemming from the SARS-CoV-2 pandemic; therefore, only four subjects completed the study and were included in the analysis. All four participants received both doses of CAP.

### 3.2. Pharmacokinetics

#### 3.2.1. Pharmacokinetic Profiles

**Figure 4:**
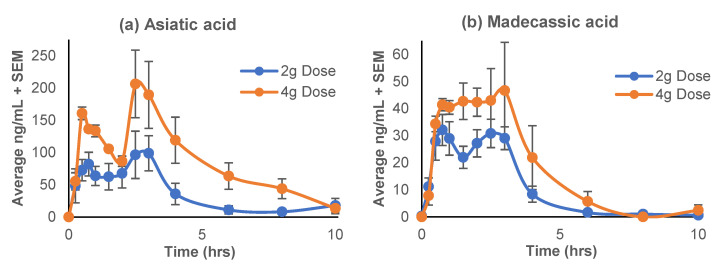
Mean plasma concentration-time profiles of the triterpene aglycones derived from *Centella asiatica* water extract product (CAP); (**a**) asiatic acid; (**b**) madecassic acid, after single oral administration of 2 g or 4 g doses in cognitively impaired older adults on cholinesterase inhibitor therapy. Data are presented as mean ± SEM (*n* = 4).

The pharmacokinetic profiles of the TT aglycones (asiatic acid and madecassic acid) are represented in the plasma concentration-time curves (Figure 4), while the pharmacokinetic parameters calculated using Excel PK-Solver software are given in Table 3. The levels of glycosidic TTs, asiaticoside and madecassoside, were below the lower limit of quantitation (LLOQ) at all time points. There was a distinct bi-modal distribution observed for both doses and both compounds; with the preliminary peak occurring at 0.5 h for asiatic acid and 0.75 h for madecassic acid, and a larger secondary peak at 2.5 h and about 3 h, respectively (Figure 4). The maximum plasma concentrations (C_max_) of asiatic acid (133–259 ng/mL) and madecassic acid (36–68 ng/mL) occurred at 2 h (T_max_) (Table 3). There was a significant two-fold difference between the C_max_ of the 2 g and 4 g doses for both aglycones (T = −6.22, *p* = 0.004; T = −2.97, *p* = 0.03). Notably, there was an approximate 2-fold difference in the AUC_0–10_ between the doses for both aglycones; however, the difference only reached significance for asiatic acid (T = −3.55, *p* = 0.02).

**Figure 5:**
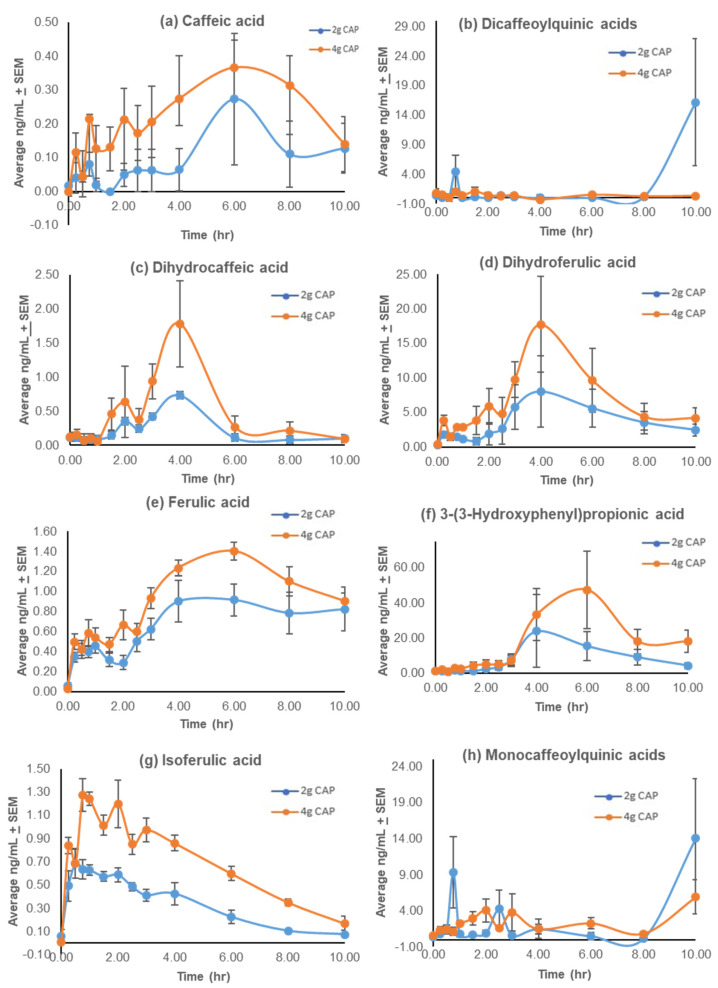
Mean plasma concentration-time profiles of the caffeoylquinic acids (CQAs) and related compounds from *Centella asiatica* water extract product (CAP) after a single oral administration of 2 g or 4 g doses in cognitively impaired older adults on cholinesterase inhibitor therapy. (**a**) Caffeic acid; (**b**) dicaffeoylquinic acids; (**c**) dihydrocaffeic acid; (**d**) dihydroferulic acid; (**e**) ferulic acid; (**f**) 3-(3-hydroxyphenyl)propionic acid; (**g**) isoferulic acid; (**h**) monocaffeoylquinic acids. Data are presented as means ± SEM (*n* = 4).

The pharmacokinetic parameters calculated using Excel PK-solver software for the mono-CQAs, di-CQAs, and their associated putative metabolites (caffeic acid, dihydrocaffeic acid, dihydroferulic acid, ferulic acid, 3-(3-hydroxyphenyl)propionic acid) (HPP), and isoferulic acid) are represented in Table 3 with plasma concentration-time profiles in Figure 5. The parent compounds, the mono-CQAs and di-CQAs were found to have a C_max_ of 7 ± 2 to 14 ± 9 ng/mL and 3 ± 0.3 to 18 ± 10 ng/mL, respectively, with no significant difference between the doses (*p* = 0.11, *p* = n/a). The T_max_ was different between the two doses and occurred at the end of the collection period (Figure 5). There was a distinct bi-modal distribution observed for both doses for dihydrocaffeic acid only, with the preliminary peak occurring at 2 h and a larger secondary peak at 4 h. All other putative metabolites had a singular distribution. Isoferulic acid had the earliest C_max_ (0.9–2 ng/mL) at 0.75 h (Table 3). The maximum plasma concentrations (C_max_) of dihydrocaffeic acid (1–2 ng/mL) and dihydroferulic acid (11–20 ng/mL) occurred at 4 h (T_max_), while the C_max_ of caffeic acid (0.3–0.5 ng/mL), ferulic acid (1–1.4 ng/mL), and 3-(3-hydroxyphenyl)propionic acid (32–42 ng/mL) occurred later, at 6 h (Table 3). There was a significant two-fold difference between the C_max_ of the 2 g and 4 g doses for dihydrocaffeic acid (T = −1.76, *p* = 0.001), dihydroferulic acid (T = −2.37, *p* = 0.05), and isoferulic acid (T = −8.50, *p* = 0.003). Notably, there was an approximate 2-fold difference in the AUC_0–10_ between the doses. This difference reached significance for caffeic acid (T = −4.04, *p* = 0.01), dihydrocaffeic acid (T = −3.02, *p* = 0.03), isoferulic acid (T = −2.26, *p* = 0.05), and 3-(3-hydroxyphenyl)propionic acid (T = −2.26, *p* = 0.05).

#### 3.2.2. Success of the Low Phytochemical Diet

Analysis of the plasma samples collected at the participants’ screening visit detected the following analytes: caffeic acid (0.8 ± 0.6 ng/mL), dihydrocaffeic acid (0.3 ± 0.2 ng/mL), dihydroferulic acid (6.6 ± 3.2 ng/mL), dicaffeoylquinic acids (0.1 ± 0.1 ng/mL), ferulic acid (1.3 ± 0.2 ng/mL), 3-(3-hydroxyphenyl)propionic acid (49.6 ± 30.4 ng/mL), isoferulic acid (2.3 ± 1.0 ng/mL), and mono-CQAs (2.5 ± 1.6 ng/mL). Significantly lower (*p* < 0.05) mean baseline values were observed at the two study visits for ferulic acid (*p* < 0.001), isoferulic acid (*p* = 0.01), and dihydroferulic acid (*p* = 0.02), while the lower values for caffeic acid (*p* = 0.08) and 3-(3-hydroxyphenyl)propionic acid (*p* = 0.06) nearly reached significance, thereby confirming the washout of the analytes of interest with the 48 h low phytochemical diet.

#### 3.2.3. Renal Excretion

**Figure 6:**
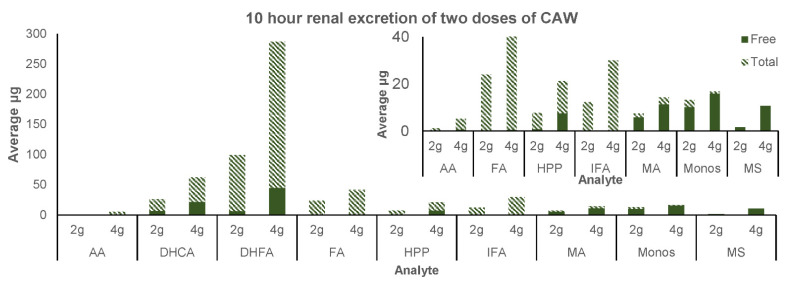
Mean urinary excretion (over 10 h) of free (unconjugated) and total (unconjugated and conjugated) forms of compounds from *Centella asiatica* water extract product (CAP) after a single oral administration of a 2 g or 4 g dose in cognitively impaired older adults on cholinesterase inhibitor therapy. AA = asiatic acid; DHCA = dihydrocaffeic acid; DHFA = dihydroferulic acid; FA = ferulic acid; HPP = 3-(3-hydroxyphenyl)propionic acid; IFA = isoferulic acid; MA = madecassic acid; Monos = monocaffeoylquinic acids; MS = madecassoside. Data are presented as means (*n* = 4).

The parent compounds asiaticoside, madecassoside, and dicaffeoylquinic acids were not detected in the screening or baseline urine (Table 4); however, small amounts of madecassoside were detected in the 10 h renal excretion of both 2 g and 4 g CAP in a predominantly free form (Figure 6). Monocaffeoylquinic acids were detected in the screening urine and the post-administration urine, predominantly in the free form but not in the baseline urine. There was a decrease in mono-CQAs in the baseline urine following the low phytochemical diet compared to screening urine; however, it did not reach significance (*p* = 0.1). Asiatic acid and madecassic acid were found in very low concentrations in screening urine, were absent in baseline urine (Table 4), and were found in very low concentrations following consumption of CAP (Figure 6). Madecassic acid was predominantly found in the free form, while asiatic acid was predominantly in the conjugated form. The putative CQA metabolites caffeic acid, dihydrocaffeic acid, dihydroferulic acid, ferulic acid, 3-(3-hydroxyphenyl)propionic acid, and isoferulic acid were detected in the screening urine, in a predominantly unconjugated form, and in the post-administration urine for both doses, in a predominantly conjugated form (Table 4, Figure 6). There was a significant decrease in the amounts of caffeic acid, dihydrocaffeic acid, ferulic acid, 3-(3-hydroxyphenyl)propionic acid, and isoferulic acid with the low phytochemical diet (Table 4). However, there were no significant differences in the renal excretion of any parent compounds or putative metabolites between 2 g and 4 g CAP over 10 h (*p* = 0.09–0.89).

### 3.3. NRF2 Activation

**Figure 7:**
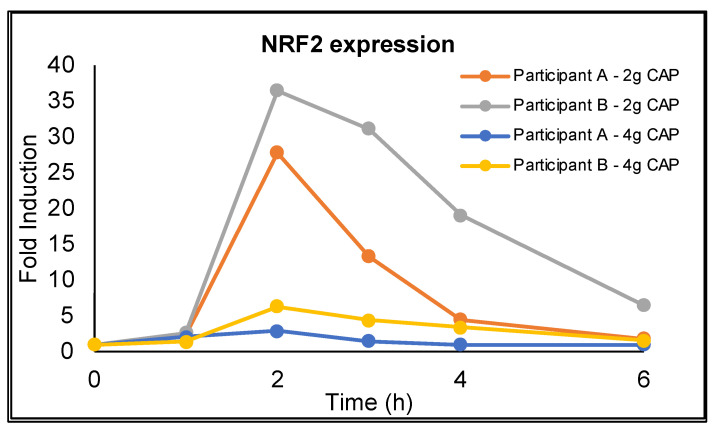
Temporal profile of induction of *NRF2* gene expression in peripheral blood mononuclear cells following oral administration of 2 g and 4 g of *Centella asiatica* water extract product (CAP) in two cognitively impaired older adults on cholinesterase inhibitor therapy. Cells were isolated using a BD Vacutainer™ CPT™ Mononuclear Cell Preparation Tube and RNA was reverse transcribed with the Superscript III First Strand Synthesis kit to generate cDNA. Relative mRNA expression was determined using TaqMan Gene Expression Master Mix and commercially available TaqMan primers for *NRF2* (*NFE2L2*) and *GAPDH*. Quantitative PCR was performed on a QuantStudio3 Machine and analyzed using the delta-delta Ct method normalizing to GAPDH expression.

*NRF2* gene expression was detected in the PBMCs of both participants where this was measured and showed a paradoxical, dose-dependent, temporal profile with peak expression occurring at 2 h after CAP administration and returning to near baseline levels by 6 h (Figure 7). The profile mirrors the pharmacokinetic profile for asiatic acid in which the T_max_ occurred at 2 h; however, the relative induction of *NRF2* gene expression for each dose does not correspond with the observed dose dependent increase in C_max_ for asiatic acid.

### 3.4. Safety and Tolerability

Both doses of CAP were well-tolerated throughout the study with no immediate or delayed adverse reactions (Table 5). No serious adverse events were reported, and no-one withdrew from the study due to the study intervention. There were no clinically significant changes in laboratory parameters (Table 5) or electrocardiograms with each dose (data not presented). The only reported adverse events that were possibly related to the study intervention were headache and dry mouth, which resolved within a seven day follow-up period.

## 4. Discussion

Pharmacokinetic and bioavailability studies form an important part of drug development, evaluating the absorption, distribution, metabolism and excretion (ADME) of bioactive molecules. The complex composition of natural products, such as botanical extracts, makes ADME studies of their numerous biologically active compounds in vivo especially difficult. However, this challenge must be met, as it has been recognized that a lack of attention to establishing the dose levels or the bioavailability of active compounds may contribute to negative results in efficacy trials of botanicals [60,61].

A growing body of preclinical evidence has established that CAW may serve as a potential treatment for cognitive impairment and AD, but translational studies are needed to assess this extract in older adults with cognitive decline. We have designed a custom *C. asiatica*-derived product (CAP) containing CAW (2 g or 4 g), along with excipients, for use in future clinical trials examining target engagement and clinical efficacy [46]. CAP is standardized to contain TTs and CQAs (the active compounds of *C. asiatica* [23]) at levels similar to those present in the CAW used in our preclinical studies. However, it is important to explore whether or not these compounds have plasma bioavailability following oral consumption of CAP by older humans and to determine their pharmacokinetic profiles to inform dosage regimens in future clinical studies. In this study, we also considered that many of the analytes of interest may be present in plasma and urine as Phase II metabolites. We, therefore, applied a hydrolytic step with glucuronide and sulfatase enzymes to all plasma and urine samples to release the compounds from any conjugated forms and to measure the total amounts. Due to resource limitations, we did not analyze plasma samples without enzyme hydrolysis to measure free, unconjugated compound. However, we were able to evaluate both the free and total analytes present in urine (Table 4, Figure 6).

### 4.1. Triterpenes

In this study, neither of the TT glycosides, asiaticoside and madecassoside, which are abundant in *C. asiatica* herb and CAP (Table 1) were detected in plasma, and only madecassoside was seen in very low amounts in the 10 h urine (1–8 µg). Asiatic acid and madecassic acid, their corresponding aglycones, which are much less abundant in CAP, were detected in both plasma and urine (Table 2, Figure 6). Similar results have been observed in human pharmacokinetic trials using pure TT compounds [42], purified TT mixtures such as CAST [62] and TTFCA [42,63], or the early version of the TT concentrated extract ECa233 [64]. In each case, only the aglycones asiatic acid and/or madecassic acid were detected in plasma, although glycosides were administered. In a more recent formulation of ECa233, however, Songvut et al. [65] included an undisclosed solubilizing agent to help with dissolution and improve bioavailability. In this study, they did detect asiaticoside (5–10 ng/mL) and madecassoside (10–13 ng/mL) in the plasma after a single administration of two separate doses (250 mg and 500 mg); however, it was substantially less than their corresponding aglycones [65]. This suggests that further dissolution is needed to facilitate absorption of the glycosides, but the aglycones remain the predominant TT absorbed by humans. In the present study, the inability to detect TT glycosides in the plasma, despite participants consuming 59–107 mg and 36–72 mg of asiaticoside and madecassoside, respectively, may be due to poor solubility, delayed absorption, variability of gastrointestinal transit time, or excretion through the feces [44,66,67]. However, the most likely explanation is in vivo bioconversion in the gastrointestinal tract to their respective aglycones via hydrolysis of the sugar moiety. Rush et al. [63] found that equimolar oral doses of asiaticoside (24 mg) and asiatic acid (12 mg) administered separately resulted in a similar 12 h total concentration (area under the curve (AUC)) of asiatic acid in human plasma. Hydrolysis of glycosides to aglycones may be performed by stomach acid or digestive enzymes or by the gastrointestinal microbiome [42], specifically a β-glycosidase-producing bacteria *Eubacterium* spp. [68]. Similarly, in dogs that were administered encapsulated aqueous extracts of *C. asiatica* standardized to asiaticoside, asiatic acid (but not asiaticoside) was identified in the plasma, peaking at 2.7 h post consumption [45]. By contrast, in mouse and rat studies, oral administration of *C. asiatica* extracts caused detectable levels of asiaticoside and madecassoside in the plasma [67,69,70,71]. A key difference between these animal models is the presence of a gastrointestinal microbiome similar to humans in the dog model but not in the rodent model [59]. Because humans have a complex gastrointestinal microbiome, we can hypothesize a significant gut microbial biotransformation of the TT glycosides into their respective aglycones.

The maximum plasma concentration (C_max_) was greater for asiatic acid (124 and 259 ng/mL) than madecassic acid (38 and 63 ng/mL) at both the 2 g and 4 g doses, respectively. This was to be expected, as there was more asiaticoside than madecassoside in the initial plant material and the standardized study product CAP (Table 1). There was a nearly 2-fold increase in C_max_ and AUC_(0–10)_ between the 2 g and 4 g doses for both aglycones, consistent with the 2-fold increase in amount of CAW consumed. This suggests that the maximum absorbable amount of the aglycones is at or above 4 g CAW daily, otherwise there would have been a non-significant difference between the two doses. The dose-dependent response found in this study is in contrast with a recent study by Songvut et al. [65] using two doses of purified TTs (modified ECa233) (250 mg and 500 mg). They found a slight increase in the C_max_ and AUC for asiatic acid and a minimal increase for madecassic acid with single dosing but a much more significant increase with repeat dosing over seven days, despite a short half-life [42]. It is hypothesized that this is a result of gradual metabolism of asiaticoside from preceding doses, due to delayed absorption or metabolism.

We observed a distinct bi-modal distribution of asiatic acid and madecassic acid, with the initial peak at 0.5 h and the second peak (T_max_) at 2.5–3 h. This pattern is congruent with the hypothesized hydrolysis of asiaticoside by intestinal enzymes prior to absorption [23] and may be explained by a prior study by Rush et al. [63], in which asiatic acid’s peak plasma concentration was reached earlier when asiatic acid was given compared to asiaticoside administration. The bi-modal absorption curve observed in this study for asiatic and madecassic acids (Figure 4) might reflect an initial absorption of the aglycones found in CAP, followed by the absorption of those released from hydrolysis of the glycosides. The higher concentration of the second peak can likely be attributed to the additive effects of enterohepatic re-circulation of these aglycones and further metabolism of the more abundant parent glycosidic compounds in the intestinal tract [56,67,69,70]. Past human studies of TTFCA [42] or asiatic acid alone [63] demonstrated that the T_max_ for asiatic acid was 4.0–4.6 h post-ingestion, which is a slightly slower absorption than seen in this study. This could be due, in part, to the difference in product preparation and dosage form. In the aforementioned studies, the participants were given the TTs in capsule form, which could add time in dissolution and metabolism, whereas our product was dissolved in warm water before consumption, allowing for more immediate absorption and metabolism. However, a different trial using the standardized *C. asiatica* TT extract product containing a solubilizing agent (ECa233) delivered in capsule form (which would suggest delayed absorption) actually demonstrated the quickest absorption rate (1.5 h) [65]. However, this group did not observe the same type of bimodal peak distribution for asiatic acid and madecassic acid Instead a large initial maximum plasma concentration peak was observed, followed by a drastically smaller one at approximately eight hours. The differences in absorption time between studies could potentially be influenced both by dosage form, race, sex, age, and by other components present in the test product, such as a solubilizing agent. For example, CAP contains a crude water extract of *C. asiatica* (CAW), as opposed to purified TT extracts used in the previous studies. As most of the *C. asiatica* preparations used in traditional medicine practice are not comprised of only purified compounds, additional investigation is needed to realize the pharmacokinetics of the TTs present in complex extracts.

There was a slight increase in the time it took to reach the maximum concentration (T_max_) and elimination half-life (T_1/2_) from 2 g to 4 g CAP, although the difference was not significant. The slight delay in peak plasma concentration at the higher dose could be due to the rate of biotransformation of asiaticoside to asiatic acid being slower at the higher CAP dose.

There was a difference between the average C_max_ and T_max_ values calculated by the PK solver software (Table 3) and the mean concentration-time profiles shown in Figure 4 for AA and MA. This apparent discrepancy is due to considerable interpersonal variability in the pharmacokinetic profiles (Supplementary material Figure S1), likely resulting from differences in gastrointestinal transit time and gastrointestinal flora influencing metabolism and absorption. When calculating the pharmacokinetic parameters, e.g., C_max_ and T_max_ in PK solver, the individual values for each participant were obtained prior to obtaining their mean value (Table 3), also allowing for the calculation of a standard error of the mean. Alternatively, if the mean plasma concentration values of all four participants at each time point are entered into PK solver, the C_max_ and T_max_ values for both AA and MA match those seen in Figure 4 (AA: 2 g C_max_ = 99 ng/mL, T_max_ = 3 h; 4 g C_max_ = 206 ng/mL, T_max_ = 2.5 h; MA: 2 g C_max_ = 32 ng/mL, T_max_ = 0.75 h; 4 g C_max_ = 47 ng/mL, and T_max_ = 3 h); however, we are unable to obtain a standard error.

### 4.2. Caffeoylquinic Acids

*C. asiatica*-specific TTs have been the predominant focus of ADME studies in vivo, with no reports to date investigating the ADME of flavonoids or CQAs following *C. asiatica* consumption. There are, however, studies of the ADME of CQAs and flavonoids stemming from other plant sources [72,73,74] and, specifically, coffee, which is rich in 3-, 4- and 5-CQA and the isomers 3,4-; 3,5-; and 4,5- di-CQA [74,75,76,77].

The metabolism of CQAs to caffeic and ferulic acid derivatives [74,75,76,77], as well as to ethyl, methyl, and vinyl catechols [78], by gastrointestinal microflora, and Phase I and II metabolism is well documented. Our data shows that many of these putative CQA metabolites are detectable in human plasma after oral CQA administration in concentrations ranging from 0.3–32 ng/mL for 2 g of CAP and 1–42 ng/mL for 4 g of CAP (Table 3). The concentration-time profiles (Figure 5) do not reveal a distinct bimodal distribution as with the TTs, but rather a small preliminary peak and a more delayed, larger single peak (T_max_) ranging from 1.3–6 h. These findings support the previous pharmacokinetic studies on CQAs derived from coffee [76] and artichoke extracts [79]. Scherbel et al. [80] reported the pharmacokinetic parameters of mono-CQAs and various metabolite groups listed previously following coffee intake, emphasizing the significant variation between subjects. Peak plasma levels were rapidly attained for the mono-CQAs and hydroxycinnamic acid metabolites (t_max_ ≤ 1 h), signifying absorption before reaching the lower intestinal tract, while the hydroxycinnamates’ dihydro metabolites reached peak levels significantly later (5.5–8.5 h), supporting their formation between the jejunum and colon. This suggests that the wide range in T_max_ observed with CAP could be due, in part, to the site in which the compounds are generated, and the small bimodal distribution could also be attributed to enterohepatic circulation [81].

Some of the pharmacokinetic curves for the mono and di-CQAs observed here are atypical, including the apparent rise in plasma concentration at the 10 h time point for some analytes. This may have been due to variability across subjects, as two participants had significantly higher values of mono-CQAs and di-CQAs at 10 h than the other two participants. One possibility is the late release of mono- or di-CQAs from more complex molecules found within CAW. Controversial data exists in the literature on the oral absorption of the CQAs [74] and is suggestive of dose-dependent absorption [75]. In humans administered coffee, intact mono- and di-CQAs were reported to be found in high levels in plasma [82,83]. Following oral administration of purified 1,5-diCQA, both intact 1,5-diCQA and its methylated metabolite, 1,5-diferuloylquinic acid, were detected in human plasma [84]. However, when artichoke extracts are administered, which also contain mono- and di-CQAs, intact CQAs were not seen in the plasma [85], and the same was observed in this study. It is hypothesized that, at higher doses, a portion of the CQAs may escape metabolism or hydrolysis due to enzyme saturation, thereby facilitating their appearance in the plasma [75].

### 4.3. Renal Excretion

The detection of madecassoside in the urine collected over 10 h post CAP administration (Figure 6) suggests that minor amounts of this glycoside may be absorbed. Asiatic acid and madecassic acid were detected in both free and conjugated forms in the urine; however, the total amounts of individual TT compounds (<20 micrograms) detected in the 10 h urine collections (Figure 6) were less than 1% of their administered amounts (Table 1). This suggests that these compounds are not excreted through the urine but, instead, are excreted via the bile and feces. For the CQAs, the di-CQA parent compounds were not detected and mono-CQAs were detected in low concentrations in the unconjugated form in the urine. This was expected, due to the very low concentration of CQAs in CAP to begin with and from prior studies using coffee, in which urinary excretion products did not include CQAs but did include CQA metabolites [76]. The metabolites were more prevalent in the urine and were found in to be predominantly in the conjugated form (Figure 6), consistent with prior coffee studies in which the metabolites were detected mostly as sulfate and glucuronide conjugates [76].

### 4.4. NRF2 Expression

Our preclinical research has demonstrated a central role for *NRF2* activation in the beneficial effects of CAW [31]. Here we demonstrate, for the first time, the activation of *NRF2* gene expression in humans following administration of a *C. asiatica* product (Figure 7). The time of maximum *NRF2* expression in PBMCs (2 h) following CAP administration corresponds with the maximum plasma levels for asiatic acid, madecassic acid, and isoferulic acid. This suggests that increased antioxidant and mitochondrial activity due to CAW may be correlated with the TT aglycones and isoferulic acid, warranting further investigation. We observed a paradoxical dose response for *NRF2* expression in PBMCs, where a stronger effect was observed with the 2 g dose than with the 4 g dose of CAP. This may be due to opposing effects on *NRF2* by some CAW components that become apparent only at the higher dose. Another possibility is the phenomenon of “hormesis”. While the extract is well tolerated and did not cause any changes in clinical measures, hormesis would be caused by a toxic factor in the extract triggering a compensatory increase in *NRF2* at low concentrations but becoming saturated at higher concentrations. However, this dose data on *NRF2* gene expression comes from only two participants so these conclusions are fairly speculative. More importantly, this data serves as a proof of concept that changes in *NRF2* expression can be detected in human subjects following administration of CAP, validating translation from prior murine studies [30,31].

### 4.5. Success of the Low Phytochemical Diet

All participants were asked to follow a diet with low phytochemical content for 48 h prior to, and for the duration of, the pharmacokinetics study visit, in order to avoid interference from dietary TT, CQA, or CQA-related compounds. A comparison of the analyte levels in the plasma and urine samples taken at the screening visit and at baseline during the two study visits (Table 4, Figure 6) confirmed the success of this approach in reducing background analyte levels. The analytes that showed the greatest difference between screening and baseline values in both the urine and plasma were ferulic and isoferulic acid. It appears that the urinary analyte levels are a better measure than plasma analyte levels when determining the effectiveness of dietary modifications.

### 4.6. Safety

*C. asiatica* is classified as a Class 1 herb by The Botanical Safety Handbook [86], meaning it can be consumed safely when correctly used. Its widespread use as a dietary supplement, the lack of reports found on the FDA CFSAN Adverse Event Reporting System (CAERS), and the available human studies support its safety [22,87,88,89]. In this study, minor adverse events were reported, including headache and dry mouth, which resolved quickly (Table 5). These events may be related to the study intervention but are more likely due to caffeine withdrawal from the low phytochemical diet. No significant changes were found in any clinical or laboratory measures (Table 2). This was also seen in prior studies using the purified TT mixture ECa233 and TTFCA taken at doses of 500 mg daily for seven days and 60 mg twice daily for 12 months [42,65]. This tolerability suggests that CAP is safe. However, future studies are needed to determine safety of repeat dosing of CAP over longer durations.

## 5. Limitations

This study was limited by its low sample size. The recruitment goal for this study was 8 participants, allowing for 20% drop out and, thus, leaving 6 evaluable subjects (see sample size, Section 2). Due to the institutional restrictions placed on clinical trials (particularly those involving vulnerable, elderly participants) because of the SARS-CoV-2 pandemic, the study was terminated with just four completing participants. However, we were still able to identify significant differences in the C_max_ and AUC between the 2 g and 4 g CAP doses (Table 3) for many of the analytes measured, with the remainder tending towards significance. Measurement of *NRF2* gene expression in PBMC was a later addition to the protocol following study initiation and could only be performed on the last two completing participants. Therefore, our findings, while consistent in the two participants, are preliminary, and our explanations for the inverse dose relationship observed are speculative. Future well-powered studies are needed to confirm these findings on *NRF2* activation by CAP. These studies should also involve the measurement of *NRF2* target antioxidant response element (ARE) genes, such as *GCLC*, *NQ01,* and *HMOX,* to confirm target engagement of this pathway. This group of ARE genes have been shown to be activated by CAW in our preclinical models reviewed earlier. Another limitation was that while this study demonstrated plasma bioavailability of TT- and CQA-related compounds from CAP, the penetration of these compounds into the central nervous system, e.g., by analysis of cerebrospinal fluid (CSF), was not evaluated. This was due to the preliminary nature of this study, where optimal timing of CSF collection after the single dosing of CAP was not known and concerns that addition of a spinal tap for CSF sampling to the protocol could limit recruitment in a study that already required invasive procedures, such as repeated blood sampling, and adherence to a strict diet for multiple days. We have previously reviewed [23] evidence for the presence of asiatic acid and madecassic acid, mono- and di-CQAs, caffeic acid, and ferulic acid in the brains of preclinical animal models following administration of *C. asiatica* or other sources of these compounds. Measurement of these CAP-derived compounds in CSF will be included in future clinical studies on CAP, which may include multiple dosing and the achievement of steady state levels in plasma and CSF.

## 6. Conclusions

This is the first study reporting the bioavailability of TTs, CQAs, and related metabolites, as well as the acute tolerability and safety following administration of a *C. asiatica* product (CAP) containing two different doses (2 g and 4 g) of *C. asiatica* water extract (CAW). Unique to this study is the use of a standardized product containing a relatively crude extract of *C. asiatica*, as opposed to the administration of a relatively pure mixture of purified or concentrated TT compounds, which has been the focus of prior pharmacokinetic literature on *C. asiatica*. The use of an aqueous extract product is more consistent with ethnopharmacological prescribing and our preclinical research. The use of a complex herb product moves away from constituent isolation, the norm in pharmaceutical development, and allows for the potential therapeutic benefit of multiple compounds with multiple targets and provides for potential synergy between compounds within the plant. In addition, all participants on this study were on cholinesterase inhibitor therapy, which was a deliberate inclusion criterion to make the data relevant to the clinical situation for individuals with mild cognitive impairment, and to account for potential pharmacokinetic interactions with the CAW components. The unknown interaction of the compounds within the plant matrix, further complicated by in vivo variations in gut microbiomes, metabolism, and the potential for drug interactions, underscores the complexity of clinical botanical medicine research.

Significant findings of this study include that TT aglycones but not glycosides are detected in the plasma and that the TTs appear to be predominantly excreted via the feces, due to low levels detected in the urine. The CQAs apparently undergo transformation in the gut and undergo phase II metabolism prior to renal excretion. This study is also the first to report preliminary evidence of increases in *NRF2* gene expression in humans after consumption of *C. asiatica*; however, well powered studies are needed to confirm this finding. Non-TT or CQA components in the plant matrix may also have bio-enhancing effects, either at the pharmacokinetic or pharmacodynamic level, which are more likely to be captured when using a complex extract as in CAP. In summary, a demonstration of the bioavailability of active TT components and CQA metabolites from CAP, as well as possible *NRF2* activation in this target human population, paves the way for future clinical studies using CAP and its evaluation as a treatment for cognitive decline.

## Figures and Tables

**Table 1:** Composition of 2 g and 4 g doses of *Centella asiatica* product (CAP).

Compound	2 g (mg/sachet ± SD)	4 g (mg/sachet ± SD)
Asiaticoside	58.98 ± 1.46	106.52 ± 4.65
Madecassoside	36.03 ± 0.48	71.93 ± 1.39
Madecassic Acid	6.29 ± 0.05	12.97 ± 0.25
Asiatic acid	5.93 ± 0.07	11.40 ± 1.02
3-*O*-Caffeoylquinic acid (Chlorogenic acid)	1.79 ±0.03	3.54 ±0.05
5-*O*-Caffeoylquinic acid (Neochlorogenic acid)	1.32 ± 0.02	2.60 ± 0.04
3,4-Dicaffeoylquinic acid (Isochlorogenic acid B)	1.03 ± 0.02	2.12 ± 0.03
4,5-Dicaffeoylquinic acid (Isochlorogenic acid C)	1.07 ± 0.04	1.92 ± 0.07
3,5-Dicaffeoylquinic acid (Isochlorogenic acid A)	0.84 ± 0.01	1.64 ± 0.03
4-*O*-Caffeoylquinic acid (Cryptochlorogenic acid)	0.59 ± 0.02	1.20 ± 0.03
1,3-Dicaffeoylquinic acid	0.45 ± 0.01	0.90 ± 0.02
1,5-Dicaffeoylquinic acid	0.36 ± 0.01	0.71 ± 0.01
Caffeic acid	0.09 ± 0.002	0.17 ± 0.003
Kaempferol	0.06 ± 0.002	0.10 ± 0.002
Naringin	0.05 ± 0.002	0.09 ± 0.002
Quercetin	0.04 ± 0.002	0.07 ± 0.003
Ferulic acid	0.03 ± 0.001	0.05 ± 0.002
Dihydrocaffeic acid	0.02 ± 0.001	0.04 ± 0.001
Rutin	0.02 ± 0.001	0.04 ± 0.002

**Table 2:** Subject demographic and baseline characteristics.

Demographic Data	Baseline	2 g CAW *	4 g CAW *
Gender, % (*n*)			
Female	40% (*n* = 2)	25% (*n* = 1)	25% (*n* = 1)
Male	60% (*n* = 3)	75% (*n* = 3)	75% (*n* = 3)
Age ^a^ (year)	70 [59]	67.8 [2.8]	67.8 [2.8]
Body mass index ^b^ (kg/m^2^)	28 ± 2	27 ± 2	27 ± 2
Systolic blood pressure ^b^ (mmHg)	132 ± 7	117 ± 5	124 ± 5
Diastolic blood pressure ^b^ (mmHg)	81 ± 3	75 ± 3	74 ± 3
Body temperature ^b^ (°C)	36.7 ± 0.08	36.6 ± 0.03	36.9 ± 0.08
Heart rate ^b^ (bpm)	68 ± 5	69 ± 3	62 ± 5
**Race, % (*n*)**			
American Indian/Alaska Native	0	0	0
Asian	0	0	0
Black or African American	0	0	0
Native Hawaiian or Other Pacific Islander	0	0	0
White	100 (5)	100 (4)	100 (4)
**Clinical laboratory screening ^b^**			
White blood cell (×10^3^/µL)	7.28 ± 0.60	NM	NM
Red blood cell (×10^6^/µL)	4.64 ± 0.21	NM	NM
Hemoglobin (g/dL)	14.3 ± 0.6	NM	NM
Hematocrit (%)	44 ± 2	NM	NM
Platelet (×10^3^/µL)	259 ± 20	NM	NM
Blood glucose (mg/dL)	81 ± 3	92 ± 6	89 ± 8
Blood urea nitrogen (mg/dL)	17 ± 3	24 ± 2 ^	24 ± 3 ^
Creatinine (mg/dL)	0.90 ± 0.06	0.85 ± 0.06	0.83 ± 0.09
Total bilirubin (mg/dL)	0.6 ± 0.1	0.7 ± 0.1	0.8 ± 0.1
Aspartate aminotransferase (U/L)	26 ± 3	28 ± 6	37 ± 2
Alanine aminotransferase (U/L)	32 ± 3	39 ± 6	43 ± 7
Alkaline phosphatase (U/L)	87 ± 12	84 ± 9	80 ± 7
Total Protein (g/dL)	7.7 ± 0.1	7.2 ± 0.2	7.1 ± 0.2
Albumin (g/dL)	3.8 ± 0.1	3.7 ± 0.1	3.6 ± 0.1
Sodium (mmol/L)	141 ± 1	141 ± 1	141 ± 1
Chloride (mmol/L)	108 ± 1	109 ± 0.5^	110 ± 1 ^
Potassium (mmol/L)	3.8 ± 0.1	3.8 ± 0.2	4.1 ± 0.3
Total CO_2_ (mmol/L)	29 ± 1	28 ± 0.3	28 ± 1
Calcium (mg/dL)	9.3 ± 0.1	9.1 ± 0.1	9.3 ± 0.9
Anion Gap	5 ± 1	5 ± 1	4 ± 1

^a^ Data are expressed as median [IQR]. ^b^ Data are expressed as mean ± SEM, (*n* = 5 for baseline, *n* = 4 for 2 g CAW and 4 g CAW). ^ = outside of reference range but deemed not clinically significant. NM = Not measured. * = Data from 10 h time point.

**Table 3:** Pharmacokinetic parameters following oral administration of *Centella asiatica* water extract product (CAP) in cognitively impaired older adults on cholinesterase inhibitor therapy.

Pharmacokinetic Parameter	Analyte	2 g CAW (*n* = 4)	4 g CAW (*n* = 4)	*p*-Value
C_max_ (ng/mL)	AA	124 ± 29	259 ± 24	0.01 *
	CA	0.3 ± 0.2	1 ± 0.1	0.23
	DHCA	1 ± 0.4	2 ± 0.2	0.001 *
	DHFA	11 ± 5	20 ± 6	0.05 *
	Di-CQAs	18 ± 10	3 ± 0.3	-
	FA	1 ± 0.2	1.4 ± 0.1	0.07
	HPP	32 ± 18	42 ± 18	0.09
	IFA	0.9 ± 0.3	2 ± 0.1	0.003 *
	MA	38 ± 3	63 ± 10	0.10
	Mono-CQAs	14 ± 7	7 ± 2	0.11
T_max_ (h)	AA	2 ± 0.6	2 ± 0.6	0.82
	CA	4 ± (2)	4 ± 1	0.40
	DHCA	3.25 ± 0.5	3.5 ± 0.5	0.20
	DHFA	3.4 ± 1	3.5 ± 0.5	0.48
	Di-CQAs	6 ± 2	0.6 ± 0.3	-
	FA	5 ± 0.6	5.5 ± 0.5	0.20
	HPP	6 ± 1.4	5.5 ± 1.7	0.32
	IFA	1.3 ± 0.6	1 ± 0.4	0.35
	MA	2 ± 0.5	2 ± 0.5	0.52
	Mono-CQAs	4 ± 2	2 ± 0.4	0.13
t_½_ (h)	AA	3.8 ± 1	2.2 ± 0.5	0.34
	CA	2.6	3.5 ± 1.2	-
	DHCA	4.8 ± 1.3	1.1 ± 0.4	0.08
	DHFA	4.5 ± 1.6	2.5 ± 0.3	0.23
	Di-CQAs	<LLOQ	3 ± 2	-
	FA	13.4 ± 5.3	7 ± 1.2	0.14
	HPP	2.3 ± 0.3	1.8 ± 0.7	0.28
	IFA	8.8 ± 5.7	2.9 ± 0.6	0.17
	MA	1.7 ± 0.5	1.7 ± 0.9	0.98
	Mono-CQAs	2 ± 0.5	3 ± 0.3	0.16
AUC (ng × h/mL)	AA	364 ± 114	935 ± 178	0.04 *
	CA	1 ± 1	3 ± 0.3	0.01 *
	DHCA	3 ± 0.4	6 ± 1	0.06
	DHFA	42 ± 18	77 ± 25	0.08
	Di-CQAs	18 ± 10	6 ± 4	0.56
	FA	7 ± 2	10 ± 1	0.21
	HPP	100 ± 46	189 ± 77	0.05 *
	IFA	5 ± 2	8 ± 0.4	0.05 *
	MA	101 ± 16	187 ± 48	0.23
	Mono-CQAs	18 ± 6	23 ± 4	0.16

Pharmacokinetic parameters were calculated using a non-compartmental analysis of plasma concentration versus time data using Excel software PK-solver (version 2.0). Two-sided paired t-tests were used to compare pharmacokinetic and clearance parameters between the 2g and 4g doses. Data are expressed as mean + SEM; CAW = *Centella asiatica* water extract; C_max_ = maximum plasma concentration; T_max_ = time to reach C_max_; AUC_0–10_ = area under the plasma concentration–time curve from time zero to time 10 h; t_1/2_ = elimination half-life; AA = asiatic acid; CA = caffeic acid; DHCA = dihydrocaffeic acid; DHFA = dihydroferulic acid; di-CQAs = dicaffeoylquinic acids; FA = ferulic acid; HPP = 3-(3-hydroxyphenyl)propionic acid; IFA = isoferulic acid; MA = madecassic acid; mono-CQAs = monocaffeoylquinic acids; * *p* < 0.05 = significant difference.

**Table 4:** Concentration of *Centella asiatica* triterpenes, caffeoylquinic acids, and their derivatives in urine samples collected at the screening visit (uncontrolled diet) and at baseline (following 48 h low phytochemical diet) on study visits.

**Analyte**	Screening Total ng/mL	Screening Unconjugated ng/mL	Baseline Total ng/mL	Baseline Unconjugated ng/mL	Screening Total: Baseline Total
**AA**	0.5 ± 5	ND	ND	ND	*p* = 0.4
**AS**	ND	ND	ND	ND	NA
**CA**	260 ± 140	184 ± 144	ND	ND	*p* = 0.02 *
**DHCA**	117 ± 64	46 ± 36	9 ± 3	0.9 ± 2	*p* = 0.03 *
**DHFA**	939 ± 507	179 ± 74	273 ± 131	8 ± 6	*p* = 0.1
**Di-CQAs**	ND	ND	ND	ND	NA
**FA**	372 ± 78	7 ± 3	17 ± 7	0.1 ± 0.1	*p* = 0.0001 *
**HPP**	91 ± 48	23 ± 14	2 ± 2	ND	*p* = 0.02 *
**IFA**	472 ± 246	4 ± 3	5 ± 2	ND	*p* = 0.02 *
**MA**	ND	ND	ND	ND	NA
**Mono-CQAs**	812 ± 749	507 ± 475	ND	ND	*p* = 0.1
**MS**	ND	ND	ND	ND	NA

Data are expressed as mean + SEM (*n* = 4); Two-sided paired t-tests were used to compare total analyte concentration between the screening and baseline samples; ND = not detected; NA = not applicable; AA = asiatic acid; AS = asiaticoside; CA = caffeic acid; DHCA = dihydrocaffeic acid; DHFA = dihydroferulic acid; di-CQAs = dicaffeoylquinic acids; FA = ferulic acid; HPP = 3-(3-hydroxyphenyl)propionic acid; IFA = isoferulic acid; MA = madecassic acid; mono-CQAs = monocaffeoylquinic acids; MS = madecassoside; * *p* < 0.05 = significant difference.

**Table 5:** Summary of adverse events of Centella asiatica water extract product after the acute administration of two different doses.

Adverse Event	2 g CAW	4 g CAW	Relation to Medication
**Psychological/General**			
Anxiety		1/4 (25%) Mild	Not related
Drowsiness	1/4 (25%) Mild	1/4 (25%) Mild	Not related
**Neurological/Muscle**			
Headache		1/4 (25%) Mild	Possibly related
Inability to sit still		1/4 (25%) Mild	Not related
Rigidity of any body part		1/4 (25%) Moderate	Not related
**Head, Eyes, Ears, Nose, and Throat**			
Blurred vision	1/4 (25%) Mild		Not related
Sore throat		1/4 (25%) Mild	Not related
Allergy symptoms	2/4 (50%) Mild		Not related
Nasal congestion/sinusitis	1/4 (25%) Mild	1/4 (25%) Moderate	Not related
**Cardiopulmonary**			
Heart racing or irregular beating	1/4 (25%)	1/4 (25%)	Not related
Hypertension/Elevated blood pressure	1/4 (25%)	1/4 (25%)	Not related
**Gastrointestinal**			
Increased appetite	1/4 (25%) Mild	2/4 (50%) Mild-Moderate	Not related
Dry mouth		1/4 (25%) Mild	Possibly related
**Genitourinary**			
Increased urination		2/4 (50%) Mild	Not related
**Skin**			
Sunburn or sensitivity of skin to light		1/4 (25%) Mild	Not related
**Whole Body**			
Generalized pain	1/4 (25%) Mild	1/4 (25%) Mild	Not related

Each event was ranked on a scale of 0–5 based upon the following criteria: 0 = absent, 1 = mild, 2 = moderate, 3 = severe, 4 = life-threatening, and 5 = fatal.

## Data Availability

The data presented in this study are available on request from the corresponding author. The data are not publicly available due to HIPAA.

## Supplementary Materials

The following supporting information can be downloaded at: https://www.mdpi.com/article/10.3390/antiox11020215/s1, Figure S1: Individual plasma concentration-time profiles of triterpene aglycones derived from *Centella asiatica* water extract product (CAP) in cognitively impaired older adults on cholinesterase inhibitor therapy.
