# Supplementary material for: Pharmacokinetics and Pharmacodynamics of Key Components of a Standardized Centella asiatica Product in Cognitively Impaired Older Adults: A Phase 1, Double-Blind, Randomized Clinical Trial
Source: Antioxidants (Basel). 2022 Jan 23;11(2):215. doi: 10.3390/antiox11020215 (PMC8868383; doi:10.3390/antiox11020215)
Supplement: Supplementary file 1 [file antioxidants-11-00215-s001.zip › antioxidants-1545495-supplementary.pdf]

Supplementary Material

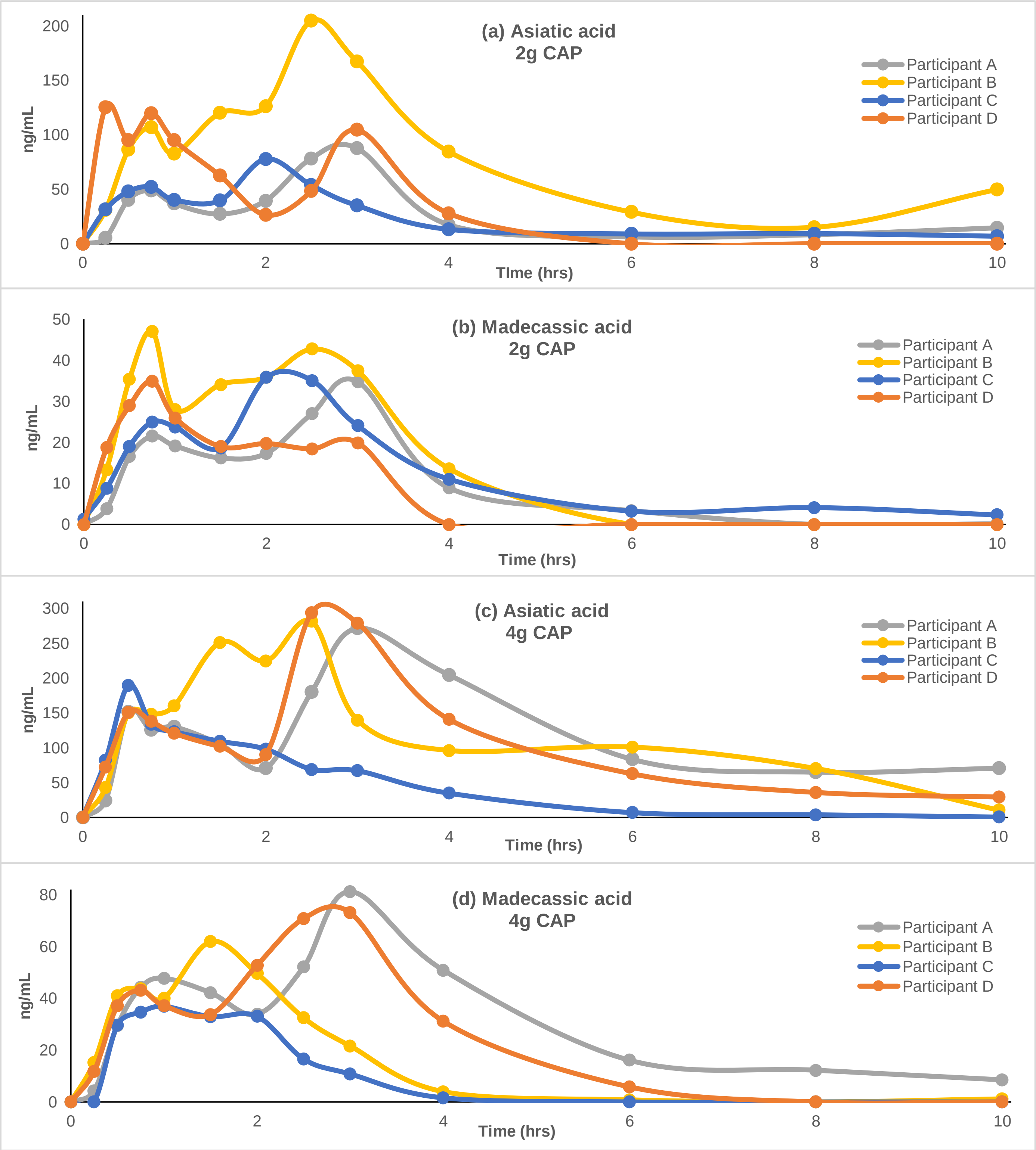

Figure S1. Individual plasma concentration-time profiles of triterpene aglycones derived from *Centella asiatica* water extract product (CAP) in cognitively impaired older adults on cholinesterase inhibitor therapy; (**a**) asiatic acid following single oral administration of 2 g CAP; (**b**) madecassic acid following single oral administration of 2 g CAP; (**c**) asiatic acid following single oral administration of 4 g CAP, (**d**) madecassic acid following single oral administration of 4 g CAP.
